# Investigation of the Temperature Effect on Electrical Characteristics of Al/SiO_2_/n++-Si RRAM Devices

**DOI:** 10.3390/mi13101641

**Published:** 2022-09-29

**Authors:** Piotr Wiśniewski, Mateusz Nieborek, Andrzej Mazurak, Jakub Jasiński

**Affiliations:** 1 Centre for Advanced Materials and Technologies CEZAMAT, Warsaw University of Technology, 02-822 Warsaw, Poland; 2 Center for Terahertz Research and Applications (CENTERA), Institute of High-Pressure Physics, Polish Academy of Sciences, 01-142 Warsaw, Poland; 3 Institute of Microelectronics and Optoelectronics, Warsaw University of Technology, 00-662 Warsaw, Poland

**Keywords:** resistive switching, RRAM, memristor, silicon oxide, MIS, MOS, temperature measurements, SCLC, TDDB, Weibull

## Abstract

In this work, we investigate the effect of temperature on the electrical characteristics of Al/SiO_2_/n++-Si RRAM devices. We study the electroforming process and show that forming voltage and time-to-breakdown are well described by Weibull distribution. Experimental current–voltage characteristics of Al-SiO_2_-(n++Si) structures are presented and discussed at different temperatures. We show that some intermediate resistance states can be observed at higher temperatures. In our analysis, we identify Space Charge Limited Conduction (SCLC) as the dominating transport mechanism regardless of the operating temperature.

## 1. Introduction

Development of modern nanoelectronics relies on technological advancement and concepts of novel devices that improve the performance of the systems. Continuous work of scientists and engineers has resulted in aggressive scaling of modern integrated circuits (IC) and performance boosters that allow maintaining progress in IC performance [1,2]. Simultaneously, a similar effort has been put into the development of memory devices which are indispensable in modern circuits. However, in order to maintain such progress, novel devices are needed. In recent years, new memory device concepts have emerged, e.g., Resistive RAM (RRAM) [3,4,5,6], Spin Transfer Torque RAM (STT-RAM) [7,8], Ferroelectric RAM (FeRAM) [9], and Phase-Change RAM (PCRAM) [10]. Resistive RAM (RRAM) has drawn attention due to its simple construction and potential to scale the device’s dimensions to achieve high density, low power, and high-speed operation. Potentially, they allow performing computation on a large amount of data in a parallel way, and in order to achieve such superior performance, different novel computing paradigms have been tested, e.g., brain-inspired computing, in-memory computing, stochastic computing, and neuromorphic computing [11,12,13]. Various oxide materials have been tested as candidates for resistive switching layers in RRAM devices [14,15,16]. Some works have presented research on SiO_2_ as a promising material for those devices [17,18,19,20]. In our recent works, we showed that well-known silicon oxide in a materials stack of Al/SiO_2_/n++-Si can also exhibit resistive switching properties [21,22]. However, little work relates to the effect of temperature on the device’s performance [23,24,25].

In this work, we investigate the effect of temperature variation on the electrical properties of devices in order to study their electrical transport mechanisms and understand their behavior. We analyze the electroforming voltage and show that it strongly depends on the temperature within a certain temperature range, whereas it saturates and does not depend on temperature for low temperatures. We show that forming voltage and time to breakdown follow the Weibull distributions and are strongly dependent on temperature. Current–voltage characteristics at higher temperature tend to vary and we can observe intermediate resistance states. We investigate and discuss the current values at ON and OFF cycles as a function of temperature and show that it is Space Charge Limited Conduction current, and that regardless of the operating temperature it remains almost the same.

## 2. Materials and Methods

**Figure 1:**
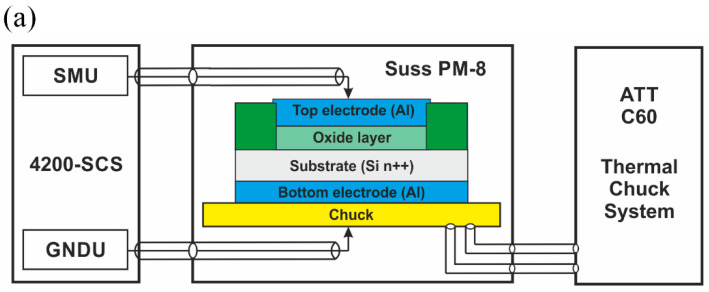

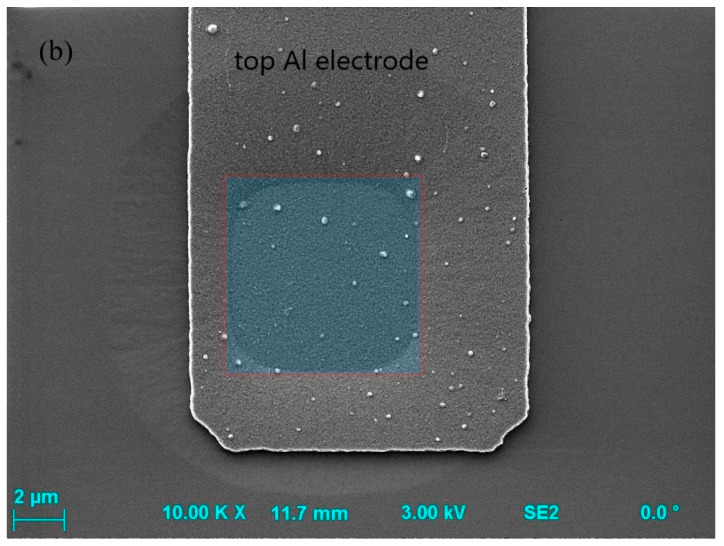
Schematic sketch of the investigated structure and measurement setup used for electrical characterization (**a**) and a top-view SEM photo of an investigated device with shaded device area (**b**).

Structures were fabricated using standard CMOS processes on a 4-inch *n*-type highly doped wafer (resistivity less than 0.005 Ω·cm). First, wafers were cleaned using the RCA method, and wet oxidation was carried out. Wet oxidation was performed in a high-temperature furnace (Thermco 2803 Furnace System) at 1000 °C, resulting in 300 nm of silicon oxide layer. Etching was performed in buffered oxide solution (BHF). Photolithography was used to define the windows for wet etching of oxide. Then a dry oxidation process was performed at 800 °C for 10 min to fabricate a thin silicon oxide layer which will serve as a resistive switching layer. It resulted in 5–6 nm thin oxide layer. Al metal electrode was sputtered and a second lithography step was performed to etch the areas that define the top electrode. In the end, the bottom electrode was fabricated on the bottom Si surface, and annealing was performed in a H_2_/Ar atmosphere. Both metal electrodes are 200 nm thick. Active area of a device is 7 × 7 µm^2^. Measurements were carried out with Keithley 4200-SCS Semiconductor Characterization System (Keithley Instruments, LLC, Solon, OH, USA) and Süss MicroTec PM8 low noise probe shield combined with ATT C60 chiller unit. The system allows controlling the measured wafer temperature within the range of −60–250 °C. DC measurements were performed using a static source-measure unit (SMU). Measurement setup and a schematic picture of the investigated device are presented in Figure 1a. In Figure 1b, we have shown a top-view SEM photo of an exemplary device.

## 3. Results and Discussion

The RRAM device generally consists of Metal–Insulator–Metal (MIM) structure. A dielectric layer is sandwiched between two metallic electrodes. Usually, the device needs an initialization through the electroforming process by applying the proper voltage. It induces soft dielectric breakdown. As a result, local defects are formed that can create a conductive filament (CF), forming a path for current flow. A current limit needs to be set by the compliance system during the forming process, e.g., through the series transistor (1T1R structure) or by the measurement setup option. The current limitation is needed to avoid the hard and destructive breakdown of the dielectric layer and control the conductive filament’s size. After forming process, the device is in a low resistance state (LRS). Conductive filament shunts the dielectric. The reset process can break CF by applying reverse polarization. The device is in the high resistance state (HRS) and can be set into the LRS when polarization is opposite to the reset operation. The set and reset operation mechanism is related to the field-driven migration of defects within the dielectric [26]. It results in the formation or opening of the CF and, therefore, a change in the resistance state. It is the so-called bipolar RRAM.

**Figure 2:**
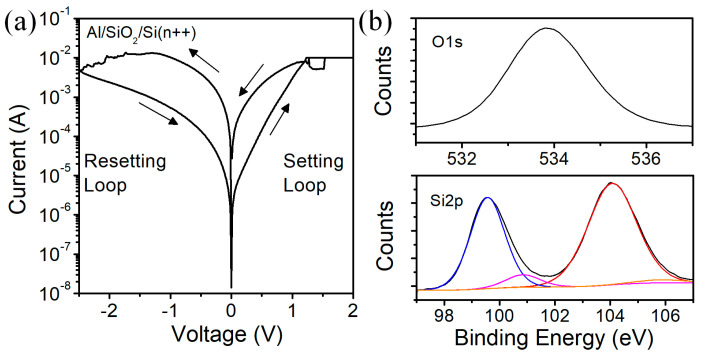
Typical I-V characteristics of a fresh Al/SiO_2_/n++-Si RRAM device measured at 25 °C (**a**) and XPS spectra (**b**) for silicon oxide used as a resistive switching layer.

In our work, we investigated Metal–Insulator–Semiconductor (MIS) Al/Silicon-Oxide/n++-Si structure. The thickness of the silicon oxide layer was measured using spectroscopic ellipsometry and resulted in 5–6 nm. Investigated structures have an area of 7 × 7 µm^2^. We have measured samples with oxide layer fabricated with the same process flow using X-ray photoelectron spectroscopy (XPS). In Figure 2, we show the typical current–voltage characteristics and obtained XPS spectra. Based on the measurements, we have calculated the oxygen (O) to silicon (Si) ratio, which resulted in O/Si in the range 1.73–1.77. This result indicates that the obtained oxide layer prior to the electroforming process has an oxygen deficiency which may result in oxide vacancies that contribute to the formation of the conductive filament during the SET cycle. Moreover, from Figure 2b on Si2p spectra we can observe that there are some silicon inclusions within the oxide layer. It has also been observed for sputter deposited silicon-oxide layers [27], and its role on resistive switching was discussed in work [28]. Hence, the conductive filament is probably a mixture of Si species and oxide vacancies.

**Figure 3:**
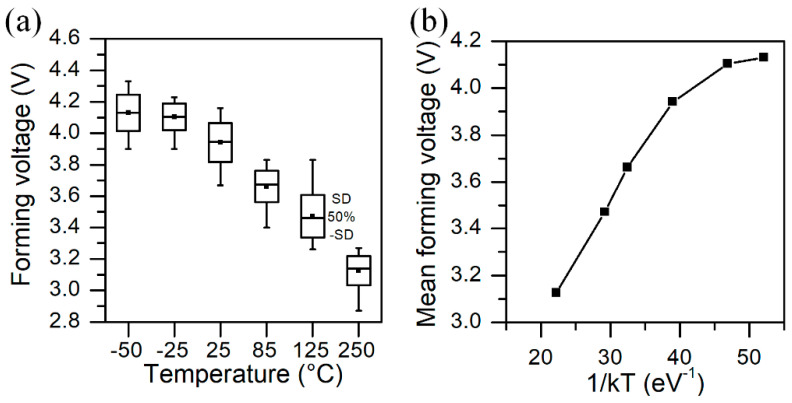
Forming voltage statistics (**a**) and mean forming voltage values (**b**) at different temperatures.

In order to understand the device behavior, we analyzed the electroforming process for various temperatures. In Figure 3, we show forming voltage statistics at different temperatures. At every temperature, we measured 30 fresh structures. We can observe that the forming voltage decreases with the increase of temperature, which agrees with theory and other experiments on breakdown phenomena [29,30]. For temperatures below 0 °C, we observe that forming voltage value saturates and is weakly dependent on temperature (down to −50 °C).

We also investigated the forming process of devices with temperature and voltage bias as parameters. Based on the measurements, the forming voltage and forming time (time-to-breakdown) t_bd_ values were extracted. The Weibull distribution had been used for oxide breakdown modeling [31,32]. Since the voltage breakdown is analogue to forming process in the RRAM switching layer, the same statistics was adopted to model forming and switching processes in resistive memory devices [33,34,35,36]. The cumulative distribution function for the Weibull distribution of statistical variable *x* is:

(1) $$F\left(x;\beta ,\lambda \right)=1-e^{-{\left(\frac{x}{\lambda }\right)}^{\beta }}$$

for *x* ≥ 0, where *β* > 0 is the shape parameter (or Weibull slope) that measures statistical dispersion and *λ* > 0 is the scale parameter, a value at which the value of statistical variable is *F* ≈ 0.63. When the Weibull plot is used for data presentation, the axes are *ln*(−*ln*(1 − *F(x*))) versus *ln*(*x*), and then the cumulative distribution function can be linearized, since

(2) W=ln(−ln(1−F(x)))=β(ln(x))−β(ln(λ))

**Figure 4:**
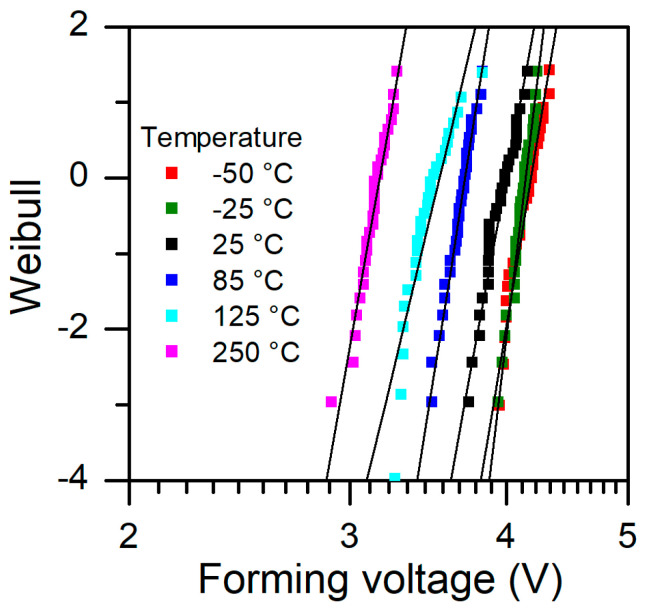
Forming voltage statistics, Weibull plot at different temperatures.

In Figure 4, we show the Weibull plot of forming voltage at different temperatures. We can observe that most of the data fit well to the Weibull statistics. Plots for low temperatures are very close, which means that there is a certain limit value that is needed to strongly affect the breakdown process. At higher temperatures, we observe some deviation from a straight line, particularly at 125 °C, where the standard deviation (SD) is the highest (see also Figure 3a). It also is visible at shape parameter of Weibull statistics, which is the smallest for extracted data. In Table 1, we show the extracted parameters of the Weibull distribution for forming voltage at various temperatures.

**Figure 5:**
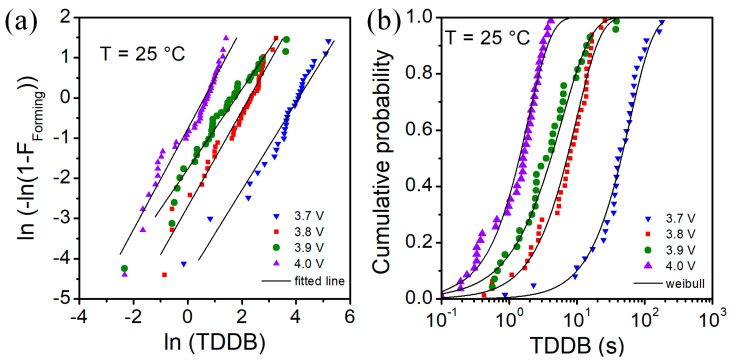
Weibull plot (**a**) and distribution (**b**) of time to breakdown for different values of voltage stress at room T = 25 °C.

**Figure 6:**
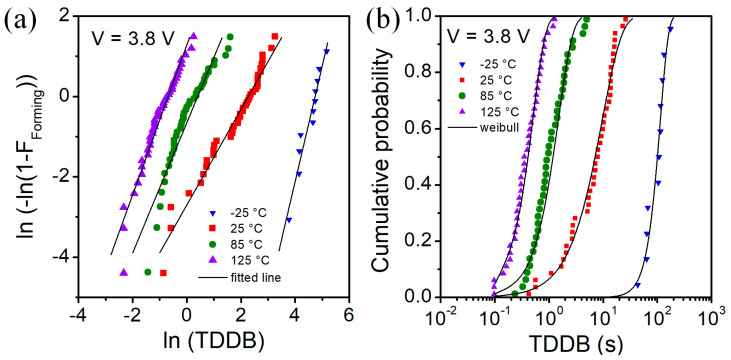
Weibull plot (**a**) and distribution (**b**) of time to breakdown for voltage stress value of 3.8 V and temperature as a parameter.

The Weibull distributions of the time-dependent dielectric breakdown (TDDB) measurements with constant voltage stress are presented in Figure 5 and Figure 6. Fits to the Weibull plots were used to extract the parameters of respective Weibull distributions. The generated Weibull distributions well match the experimental data as shown in Figure 5b and Figure 6b.

In Table 2, we show Weibull distribution parameters for time-dependent breakdown for forming process of investigated RRAM devices at various temperatures and different stress voltages. Forming time (t_bd_) decreases with the increase of forming voltage and temperature. Scale factor changes with the temperature. For V = 3.8 V, we can see that at 85 °C the scale factor is ~6 times lower than at 25 °C, whereas at 125 °C it is ~20 times lower. In general, the effect of voltage stress value is weaker than the effect of temperature on forming time. As we can observe at T = 85 °C, the parameters of the distributions of TDDB for several stress voltage values are very close. The Weibull slope β is within range 1–3 for investigated voltage stress values and temperatures.

**Figure 7:**
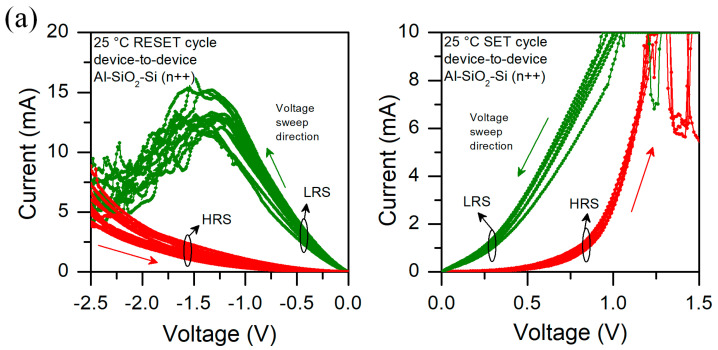

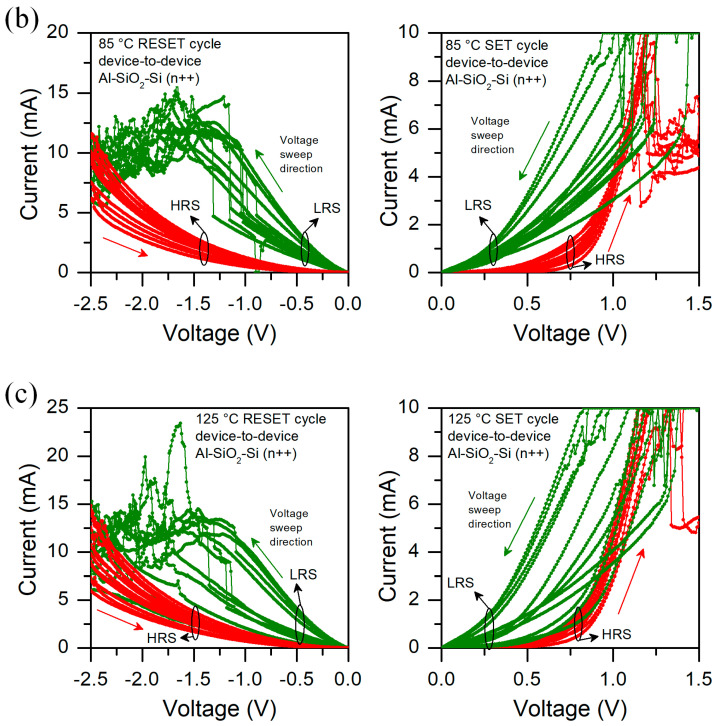
I-V characteristics of Al/SiO_2_/n++-Si RRAM device measured at (**a**) 25 °C, (**b**) 85 °C, and (**c**) 125 °C for six different structures. Arrows indicate the voltage sweep direction, LRS is marked in green color, whereas HRS in red color.

**Figure 8:**
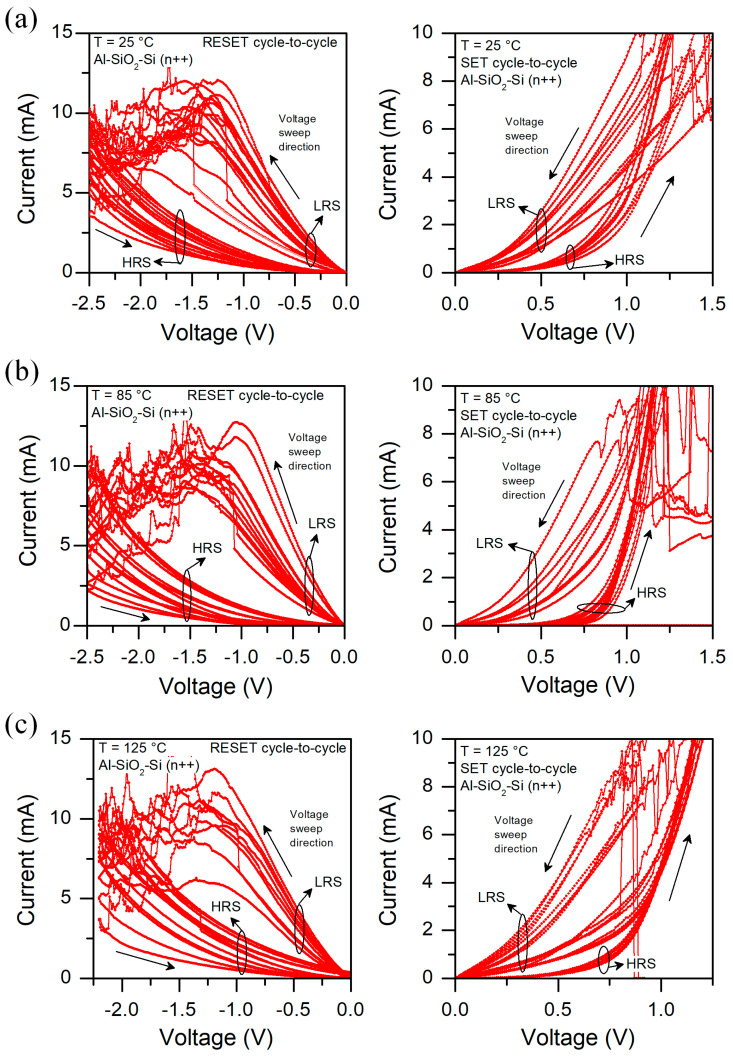
I-V characteristics of Al/SiO_2_/n++-Si RRAM device measured at (**a**) 25 °C, (**b**) 85 °C, and (**c**) 125 °C showing cycle-to-cycle variability. Arrows indicate the voltage sweep direction.

We have also analyzed the current–voltage characteristics at different temperatures. In Figure 7, we show the I-V curves for a few structures measured at 25, 85, and 125 °C in a sequence. We can observe that at 25 °C devices behave in a similar way. We can easily distinguish between High Resistance State (HRS) and Low Resistance State (LRS) for SET and RESET cycles. During SET cycle, we can observe that after reaching the compliance current (CC) level, sometimes current drops abruptly and then returns to its previous level. When a CC level is obtained, the measurement unit works as a current source. It may cause a partial filament dissolution due to local heating; therefore, CF is broken which results in a current drop. After that, as voltage sweep is continued and an electric field induces filament formation. Those instabilities may be related to the relatively high current value passing true devices in CC mode, resulting in local Joule heating [37]. At 85 °C we notice that the HRS is slightly different for different structures during the SET cycle, whereas for LRS we observe some intermediate resistance states (IRS). We also observe current drops for HRS above 1.0 V. In the RESET cycle, HRS current varies significantly, whereas at LRS we can see sudden current rises. In RESET cycle, when a structure is in IRS and the voltage is big enough, we sometimes observe an abrupt switch to LRS. At 125 °C all those effects are even more pronounced, and variability is higher. In Figure 8, we present the measurement showing cycle-to-cycle variability at different temperatures. We can observe that temperature affects the HRS state, in particular for RESET cycle. Those results indicate that thermal effects have a pronounced effect on the device’s performance and may cause partial filament formation/disruption [29].

**Figure 9:**
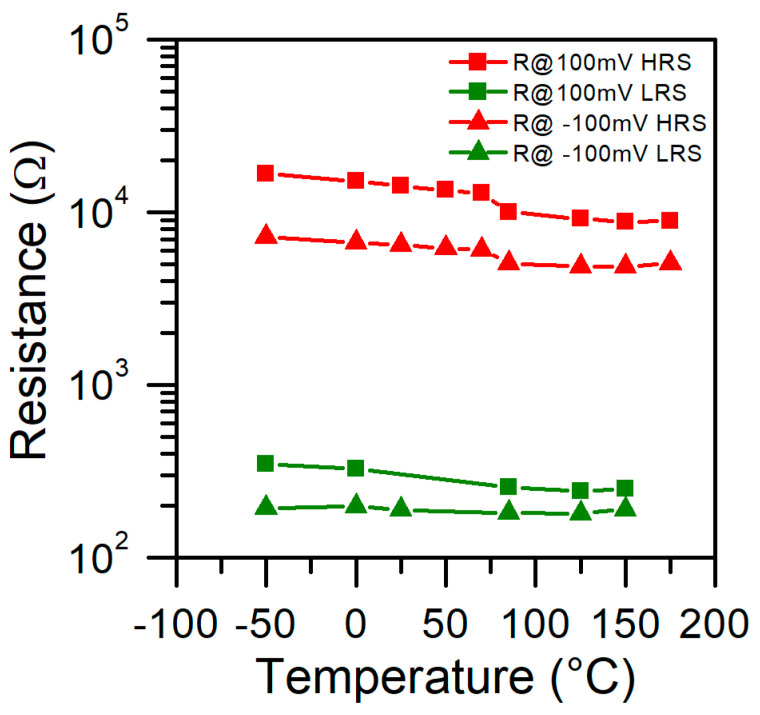
Differential resistance value of Al/SiO_2_/n++-Si RRAM device measured at various temperatures and V = ±100 mV for HRS and LRS.

In Figure 9, we show the differential resistance value of the Al/SiO_2_/n++-Si RRAM device measured at various temperatures and voltage ± 100 mV for HRS and LRS. In order to not affect the structure’s state by sweeping the voltage within wide range, we turned off the device and then measured the I-V for low voltages (<0.5 V) for different temperatures subsequently. Similarly, we carried out measurements for LRS after setting on the device. Resistance for HRS and LRS differs between polarizations due to the asymmetry of the current–voltage characteristics. Nevertheless, the trend is similar. We found that resistance decreases with temperature, which is a semiconductor-type relation of R vs. T function. In LRS, this dependence is relatively week or not visible, whereas in HRS it is stronger. For higher temperatures, we observe a change in the slope of the curve and a sudden decrease in resistance. A possible explanation of such behavior is that in HRS, the CFs paths are broken, and there is a gap that restricts the current flow. When temperature rises, the CF thermal expansion causes the shrinkage of the gap. In turn, it results in a higher current and a decrease of resistance. In LRS, a gap is closed so CF filament is already formed. However, in um size structures, there might be many CFs, and some of them may not be formed. Some of them might be partially broken, so increased temperature also results in decreased resistance. 

**Figure 10:**
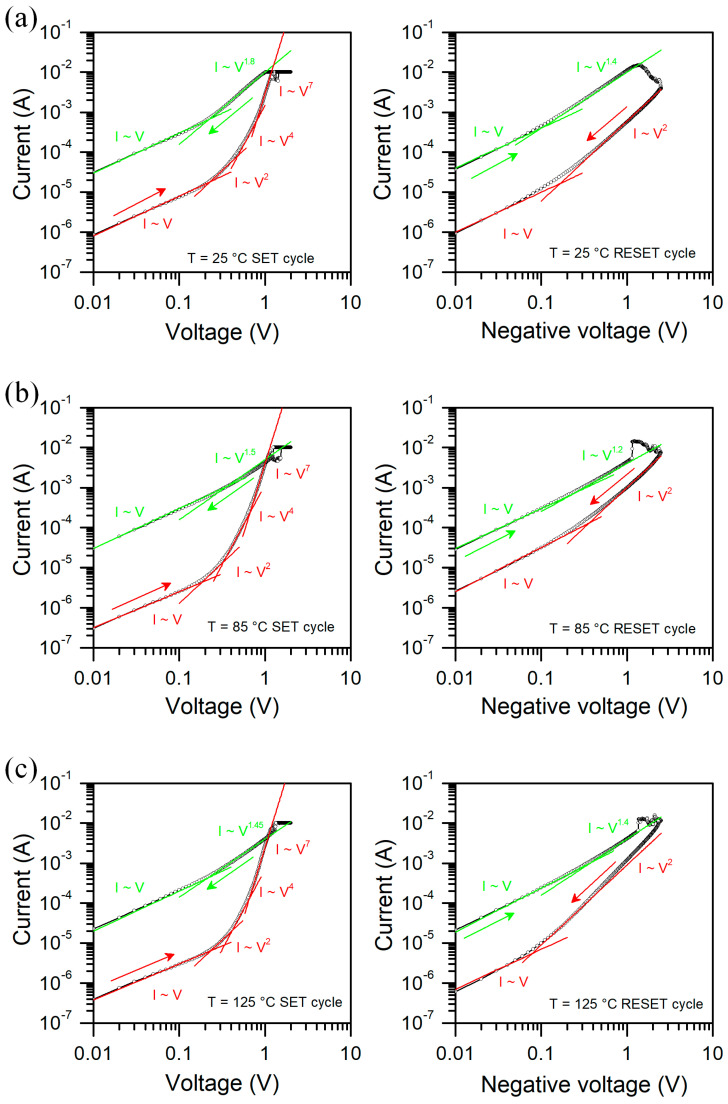
Current-voltage characteristics of Al/SiO_2_/n++-Si RRAM device with fitted slope curves at various temperatures, (**a**) 25 °C, (**b**) 85 °C and (**c**) 125 °C.

In Figure 10, we show current–voltage characteristics of the Al/SiO_2_/n++-Si RRAM device with fitted slope curves at various temperatures. We observe Space Charge Limited Conduction (SCLC) at HRS for both polarizations, regardless of the measurement temperature. For SET cycle at low voltages, current is proportional to the voltage, then we have a quadratic and higher order dependence I~V^m^, where m = 4 and 7. SCLC is related to the transport through a region containing traps. Depending on the trap states energetic distribution we can obtain different slopes of curve [38]. For RESET cycle we observe only parabolic dependence at HRS. At LRS, we observe linear dependence for low voltages and I~V^m^ with m between 1.2 and 1.8, which may indicate some randomly distributed traps due to incomplete formation or local rupture of conductive filaments. Nevertheless, regardless of the temperature, the transport type remains the same. Current level may vary with temperature, but slopes in different regions of the I–V curve almost do not change.

## 4. Conclusions

This work investigates the temperature effect on the electrical characteristics of SiO_2_-based Metal–Insulator–Semiconductor RRAM devices. We analyzed the electroforming process and current–voltage characteristics of Al/SiO_2_/n++-Si structure and identified the transport mechanisms. It is mainly Space Charge Limited Conduction related to the transport through traps within the oxide layer. Electroforming voltage and time-to-breakdown follow the Weibull distribution, which can be used to analyze the statistical properties. We discuss the effect of temperature on resistance at low voltages. Our work shows that temperature has a pronounced effect on the I-V characteristics of investigated devices.

## Figures and Tables

**Table 1:** Weibull distribution parameters for forming voltage of investigated RRAM devices at various temperatures.

Temperature (°C)	Shape Parameter *β*	Scale Parameter λ (V)
−50	43.57	4.18
−25	59.93	4.14
25	38.95	4.00
85	45.30	3.71
125	30.01	3.54
250	40.99	3.17

**Table 2:** Weibull distributions for time-dependent breakdown for forming process of investigated RRAM devices at various temperatures and different stress voltages.

Temperature (°C)	Stress Voltage (V)	Shape Parameter *β*	Scale Parameter λ (s)
25	3.7	1.09	60.16
25	3.8	1.19	9.65
25	3.9	0.99	5.93
25	4.0	1.25	1.84
−25	3.8	2.88	113.78
85	3.8	1.62	1.49
85	3.9	1.24	1.12
85	4.1	1.20	1.03
125	3.8	1.86	0.50

## Data Availability

The data presented in this study are available on request from the corresponding author.
